# Crusted scabies at a tertiary care center: Case series and cautionary tale

**DOI:** 10.1016/j.jdcr.2023.08.030

**Published:** 2023-09-09

**Authors:** Michael Lause, Karissa Libson, Abraham M. Korman, Nora Colburn, Shandra Day, Marek Greer, Michele Hardgrow, Kimberly Malcolm, Marcy Mcginnis, Elizabeth Seely, Justin Smyer, John Trinidad

**Affiliations:** a Department of Dermatology, The Ohio State University Wexner Medical Center, Columbus, Ohio; b Ohio State University College of Medicine, Columbus, Ohio; c Department of Infectious Disease, Internal Medicine, The Ohio State University Wexner Medical Center, Columbus, Ohio; d Occupational Health and Wellness, The Ohio State University Wexner Medical Center, Columbus, Ohio; e Department of Clinical Epidemiology, The Ohio State University Wexner Medical Center, Columbus, Ohio; f Department of Dermatology, Massachusetts General Hospital, Harvard Medical School, Boston, Massachusetts

**Keywords:** crusted scabies, epidemiology, infectious diseases, medical dermatology, occupational health, prevention, public health, scabies

## Introduction

Scabies is a cutaneous infection characterized by rash and pruritus that is caused by the burrowing of *Sarcoptes scabiei* mites in the epidermis. Crusted scabies is the most severe form of this infestation and typically occurs in patients with risk factors including immunosuppression, cognitive impairment, advanced age, and immobility. Crusted scabies is highly contagious and has the potential to cause significant outbreaks in patients in close living quarters; in the hospital setting, it has the potential to cause significant morbidity to other patients and hospital staff.^1^ In addition, management of nosocomial scabies outbreaks may require substantial administrative and financial resources. Herein, we describe the presentation, diagnosis, and treatment of 3 patients with crusted scabies in a tertiary care hospital; further, we highlight the broad sweeping clinical and economic impacts these infections had on the medical center.

## Case series

### Case 1

**Fig 1:**
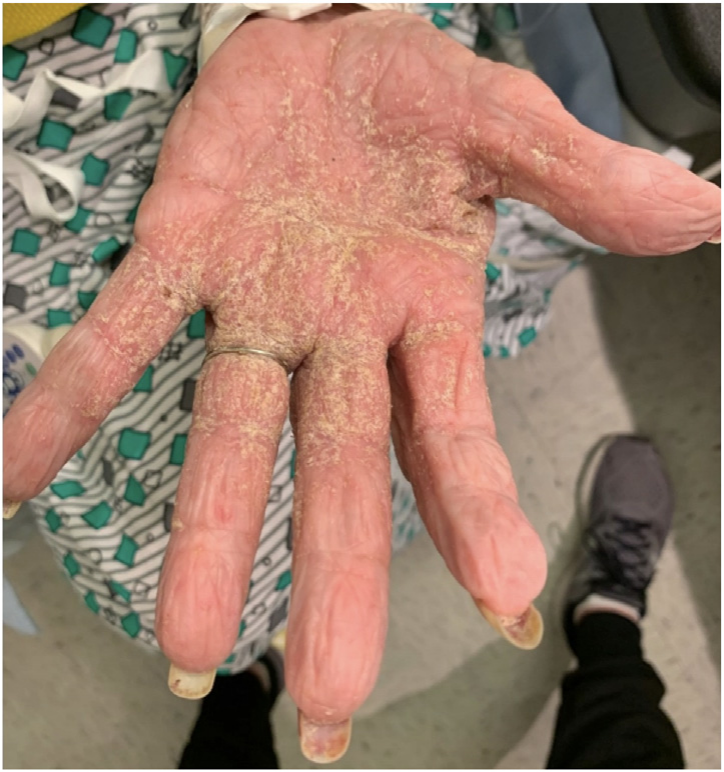
Case 1: Extensive hyperkeratosis of the palm and interdigital web spaces.

**Fig 2:**
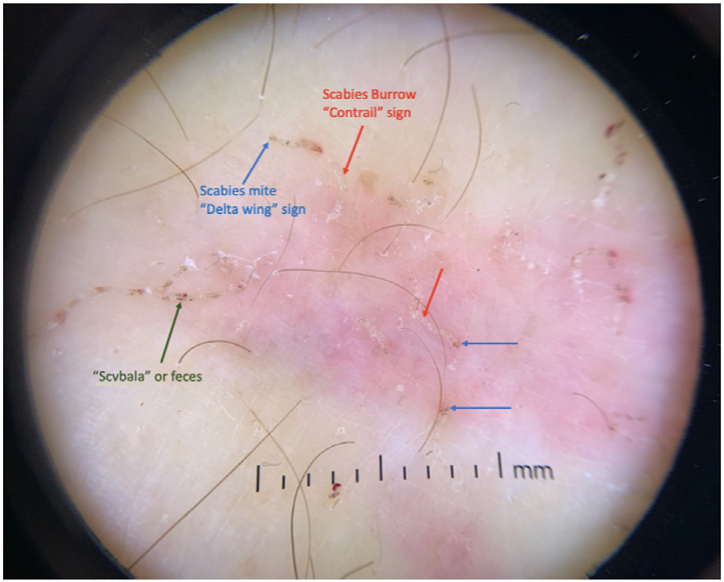
Case 1: Positive “delta wing” and “contrail” signs confirmed a diagnosis of crusted scabies. Blue arrows: scabies mite; red arrow: scabies burrow; black arrow: scybala or feces.

**Fig 3:**
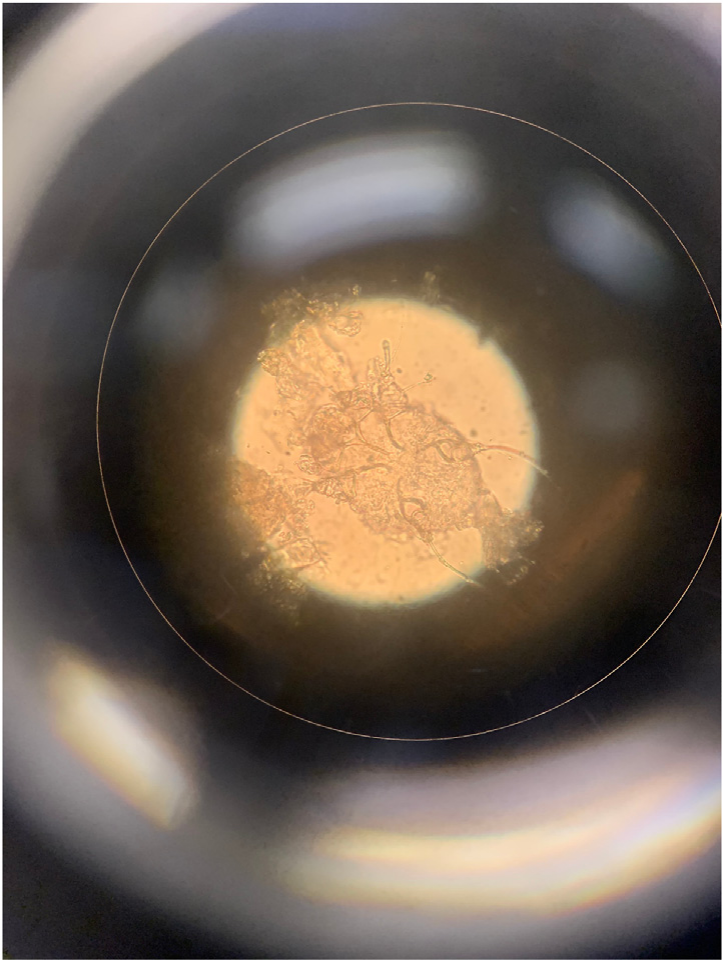
Case 1: *Sarcoptes scabiei* mite visualized with mineral oil preparation under light microscopy.

An elderly woman with a history of advanced Alzheimer disease was transferred from an extended care facility to our institution with a longstanding rash previously treated with topical corticosteroids and oral antihistamines. Dermatology was consulted, and examination revealed hyperkeratotic and excoriated papules and plaques on the face, trunk, and extremities with extensive hyperkeratosis of the palms and interdigital web spaces (Fig 1). Crusted scabies was suspected and contact precautions were immediately implemented. Dermatoscopy revealed “delta wing” and “contrail” signs, and mineral oil skin scrapings showed numerous live scabies mites on light microscopy (Figs 2 and 3). Epidemiology was notified for contact tracing purposes. Per Ohio Department of Public Health guidelines, the patient completed a course of combined topical permethrin (5% cream with full-body application repeated daily for 7 days, then twice weekly for an additional 3 weeks) and oral ivermectin (200 μg/kg/dose on days 1, 2, 8, 9, and 15).^2^ Following resolution with treatment at our institution, the patient was discharged to an extended care facility.

### Case 2

An elderly man with history of heart failure, chronic kidney disease, and vascular dementia was brought to the emergency department for sepsis and acute encephalopathy. On examination, the primary team noted a diffuse rash that had been diagnosed as a morbilliform eruption secondary to hydralazine 4 months prior. At the time of that diagnosis, hydralazine was stopped and the patient started a 2-week course of topical steroids; however, the rash persisted for months until hospitalization at our institution.

**Fig 4:**
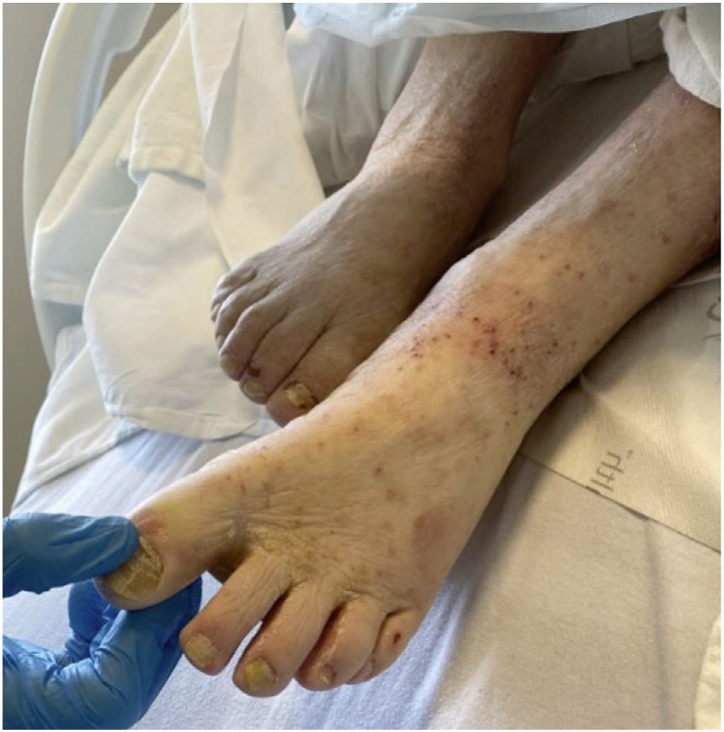
Case 2: Excoriated and erythematous papules and erosions on the dorsal foot with hyperkeratosis of the interdigital web spaces.

**Fig 5:**
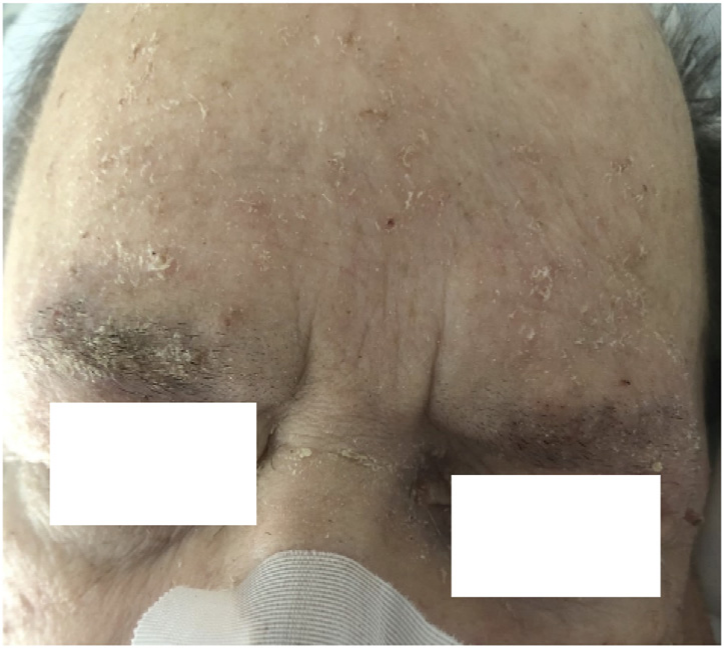
Case 2: Mildly erythematous and hyperkeratotic papules accentuated at the eyebrows.

Upon inpatient dermatology evaluation, the patient was noted to have diffuse erythematous, excoriated, and hyperkeratotic papules and erosions, which were accentuated at the umbilicus, waist, interdigital spaces, and eyebrows (Figs 4 and 5). Extensive cutaneous burrows were noted on gross examination of acral surfaces, and dermatoscopy revealed positive “delta wing” and “contrail” signs, confirming a diagnosis of crusted scabies. Bedside staff and epidemiology were notified, contact precautions were instituted, and the patient completed topical permethrin and oral ivermectin per Ohio Department of Public Health guidelines. The patient’s hospital course was complicated by septic shock with methicillin-resistant *Staphylococcus epidermidis*, attributed in part to crusted scabies infection. Following stabilization of the patient’s complex medical diseases and treatment of crusted scabies, the patient was discharged with home health services.

### Case 3

**Fig 6:**
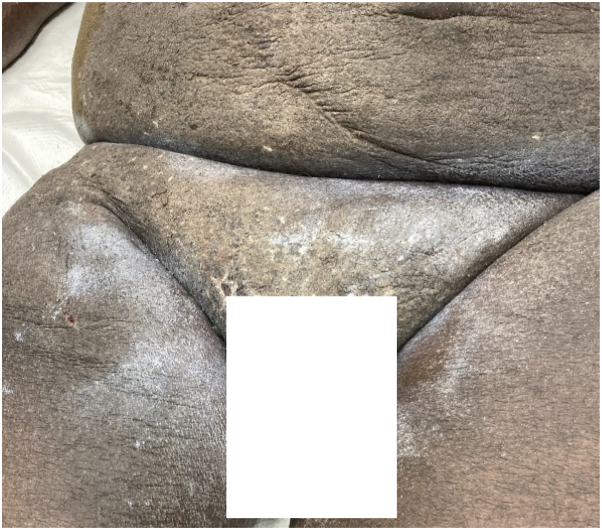
Case 3: Diffuse and extensive hyperkeratosis and lichenification of the groin, inner thighs, and lower abdomen.

**Fig 7:**
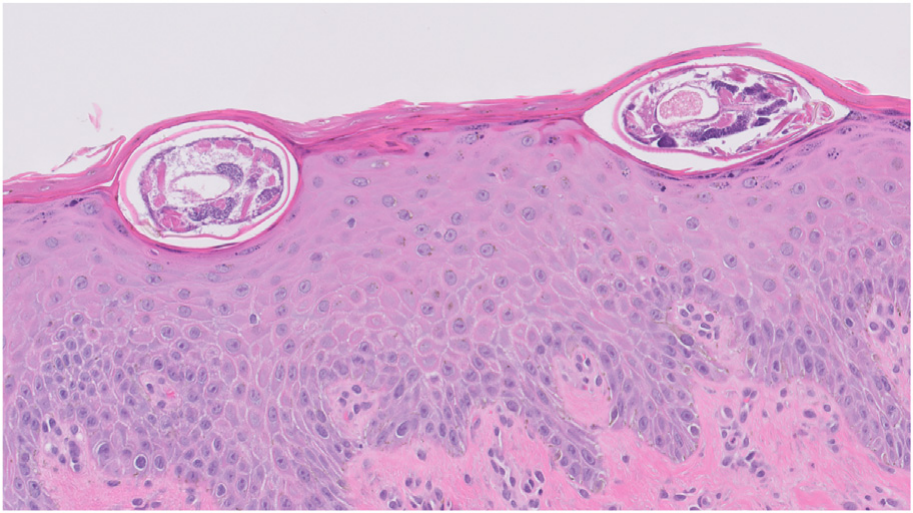
Case 3: *Sarcoptes scabiei* mites observed within the epidermis. (hematoxylin and eosin stain; original magnification : ×100.)

A middle-aged man with history of kidney transplant, on oral mycophenolate mofetil and tacrolimus was brought to the emergency department for septic shock and acute hypoxic respiratory failure. On primary team examination, the patient was noted to have coarse abdominal skin and was treated with emollients for xerosis cutis. Later in the hospital admission, dermatology was consulted for persistent rash, and examination demonstrated extensive xerosis and lichenification, with the skin having the appearance of “wet sand” (Fig 6). A diagnosis of crusted scabies was confirmed with positive “delta wing” and “contrail” signs on dermatoscopy and microscopic visualization of live scabies mites with mineral oil skin scraping. Scabies mites were also seen on histology from biopsy of a suspicious nodule, which was performed to rule out infection (Fig 7). Contact precautions were instituted, and the patient completed treatment with topical permethrin and oral ivermectin per Ohio Department of Public Health guidelines. His hospital course was complicated by polymicrobial sepsis without a clear source and multisystem organ failure. Following resolution of these conditions, the patient was discharged to inpatient rehabilitation services.

## Discussion

Crusted scabies is a rare and severe presentation of scabies characterized by the presence of millions of *Sa scabiei* mites. The increased mite burden in crusted scabies compared with noncrusted scabies predisposes to increased disease transmission through not only skin contact but also environmental infestation.^1^ In health care settings, scabies outbreaks typically stem from patients with undiagnosed crusted scabies who exemplify well-known risk factors, such as cognitive impairment, immunosuppression, advanced age, and immobility (Table I). Prompt suspicion and diagnosis of crusted scabies are critical to preventing outbreaks in nosocomial settings. In 2 of the described cases, patients demonstrated multiple risk factors and had a history of persistent rash for months before admission. Misdiagnosis is common in crusted scabies, as it is often mistaken for psoriasis, drug reactions, and acral eczema.^3^, ^4^, ^5^ Additionally, patients are often misdiagnosed and prescribed topical corticosteroids, which can lead to and worsen crusted scabies infections.^5^

**Table I:** Risk factors of crusted scabies seen in each case

Case	Cognitive impairment	Immunosuppression	Elderly (age >65 y)	Immobile
case 1	x		x	x
case 2	x		x	x
case 3		x		

Morphologic findings in crusted scabies can be diverse but often include some combination of crusted or psoriasiform plaques, hyperkeratosis, excoriations, and/or burrows.^6^ The distribution of mites is often multifocal and has a tendency toward body surfaces containing a thin epidermis; the most common locations are periumbilical skin, areolae, axillary folds, waist, genitals in men, and interdigital web spaces.^7^^,^^8^

The diagnosis of crusted scabies can be confirmed by direct visualization of scabies mites, eggs, or feces using any one of the following modalities: light microscopy with mineral oil preparation, histology, or dermatoscopy. On histology, scabies mites are typically seen within the epidermis, as the mites are unable to penetrate the dermoepidermal junction.^6^ On dermatoscopy, imagery of an airplane and its contrails is used to depict the appearance of scabies mites and burrows, respectively. More precisely, the “delta wing” sign describes the triangular head of the scabies mite, which is likened to an airplane; the “contrail sign” represents the trailing burrow, which is compared with an airplane’s trailing vapor streams often observed in the sky (Fig 2). As the infection clears, so too does the presence of the delta wing sign as the mites decompose.^9^^,^^10^

Unrecognized and untreated crusted scabies frequently leads to outbreaks in family homes, hospitals, and places of communal living such as extended care facilities.^3^^,^^4^ Scabies outbreaks in health care facilities can lead to significant morbidity for other patients and staff, in addition to administrative burden.^11^ In all 3 cases, the patients arrived at the hospital without a diagnosis of scabies and did not have contact precautions, leading to numerous exposures for frontline health care workers. Once scabies was suspected, patients were placed on contact precautions and hospital epidemiology was notified. Once notified, an exposure investigation was initiated in collaboration with Occupational Health and pharmacy. An exposure was defined as providing direct patient care without wearing a gown and gloves. Contact tracing utilized medical and staffing records for each case and identified 373 total employee exposures. At the hospital outpatient pharmacy, exposure was confirmed via a standard questionnaire assessing the temporal relationship and degree of exposure. Prophylactic oral ivermectin or topical permethrin were provided to all individuals with confirmed exposure in accordance with Ohio Department of Public Health guidelines. The hospital’s outpatient pharmacy then provided a list of all exposed and treated individuals to Occupational Health for follow-up. An associated direct cost for the medications was estimated to be over $10,000. Additionally, there were numerous indirect costs, including the implementation of a large-scale contact tracing protocol, frequent room sanitization and linen changes, and pharmacist and administrative time, all of which contributed to the organizational costs and challenges to the medical center. Following implementation of epidemiology protocols, including contact tracing and prophylactic treatment for exposed health care workers, no additional patients or staff became infected in any of the cases.

Patients with crusted scabies are at risk for life-threatening and long-term sequelae of scabies, namely secondary bacterial infections.^6^ Two of the patients in this series experienced infections that likely had contributions from the underlying crusted scabies infestation: patient 2 with methicillin-resistant *St epidermidis* septic shock and patient 3 with polymicrobial sepsis and multisystem organ failure.

## Conclusion

In this case series, we describe the risk factors, presentation, diagnostic features, and clinical course of 3 patients diagnosed with crusted scabies at a tertiary care medical center. We demonstrate the magnitude of morbidity and administrative burden that undiagnosed crusted scabies infections had on our hospital; in particular, these cases led to 373 total employee exposures, with the cost of prophylactic ivermectin and permethrin exceeding $10,000. Ultimately, scabies should be kept on the differential diagnosis list for pruritic rashes in patients who exemplify well-known risk factors, and contact precautions should be instituted while diagnostic workup is pursued.^11^

## Conflicts of interest

Dr Trinidad is on the editorial board of JAAD. Drs Lause, Korman, Colburn, Day, and Greer and authors Libson, Hardgrow, Malcolm, Mcginnis, Seely, and Smyer have no conflicts of interest to declare.
